# CLRM-09. INCORPORATING EXTERNAL CONTROL ARM IN MDNA55 RECURRENT GLIOBLASTOMA REGISTRATION TRIAL

**DOI:** 10.1093/noajnl/vdab112.008

**Published:** 2021-09-21

**Authors:** Ruthanna Davi, Antara Majumdar, Martin Bexon, Nicholas Butowski, Chandtip Chandhasin, Melissa Coello, Sunit Das, Fahar Merchant, Nina Merchant, David Reardon, Martin Roessner, John Sampson

**Affiliations:** 1 Medidata Acorn AI, New York, NY, USA; 2 Medicenna Therapeutics Inc, Toronto, ON, Canada; 3 University of California San Francisco, San Francisco, CA, USA; 4 Dana-Farber Cancer Institute, Boston, MA, USA; 5 Parexel International, Newton, MA, USA; 6 Duke University Medical Center, Durham, NC, USA

## Abstract

**BACKGROUND:**

Drug development in recurrent glioblastoma multiforme (rGBM) is challenging. For randomized controlled trials (RCTs) short survival horizons and limited life-prolonging treatment options may delay accrual and introduce bias through differential dropout of control patients. Comparing results of a single-arm Phase 2b trial of intratumoral delivery of MDNA55 (an interleukin-4 receptor targeted fusion protein) to an external control arm, we sought early efficacy insights and consideration by the FDA of incorporating an ECA in a Phase 3 registrational trial.

**METHODS:**

Using propensity score weighting, we compared rGBM patients from the Phase 2b trial (NCT02858895) (2017-2019) to patients from rGBM registries who had received standard of care therapies (2011-2019) and met eligibility requirements. Propensity scores were estimated using a logistic regression model with 11 covariates. We compared the propensity score weighted groups according to demographic and disease attributes before and after weighting and compared overall survival between the two groups.

**RESULTS:**

Through propensity score weighting, 43 (98%, 43/44) MDNA55 patients and 40.80 weighted ECA patients (from 62 unweighted registry patients) were identified for comparison. MDNA55 and ECA patients were balanced on all baseline characteristics (i.e., standardized mean difference ≤ 0.15). Compared to ECA patients, MDNA55 patients had a 37% lower hazard of death (hazard ratio 0.63, 95% confidence interval: 0.39,1.02).

**CONCLUSION:**

In advance of a Phase 3 trial, comparison of Phase 2b trial results to an ECA suggests that MDNA55 may be efficacious in rGBM. In view of the known challenges associated with drug development for rGBM, these results provided a proof-of-concept for the design of a novel hybrid Phase 3 trial. This planned Phase 3 trial incorporates propensity score weighting to create a composite hybrid randomized and external control arm, an approach preferred by the FDA over full replacement of a randomized control with an external control.

